# Design, power, and alpha levels in randomized phase II oncology trials

**DOI:** 10.1016/j.esmoop.2022.100779

**Published:** 2023-02-01

**Authors:** A. Haslam, T. Olivier, V. Prasad

**Affiliations:** 1 Department of Epidemiology and Biostatistics, University of California San Francisco, San Francisco, USA; 2 Department of Oncology, Geneva University Hospital, Geneva, Switzerland

**Keywords:** clinical trials, sample size calculations, reporting

## Abstract

**Background:**

The statistical plan of a phase II trial should balance minimizing the premature termination of potentially beneficial therapies (i.e. false negatives) and the further, costly testing of ineffective drugs (i.e. false positives). We sought to examine the methodology, reporting, and bias in the interpretation of outcomes of phase II oncology trials in recent years.

**Materials and methods:**

In a retrospective cross-sectional analysis, we reviewed all full-length articles published on PubMed from 1 January 2021 to 20 June 2022. We searched for data regarding the sample size calculation (number, α value, power, and expected effect size), the primary and secondary outcomes and results, and the authors’ conclusion of the study.

**Results:**

About 5.4% of studies (*n* = 10) used a statistical power that was inferior to 80%, and 16.7% (*n* = 34) did not indicate the level of power for the sample size calculation. Approximately 16.7% (*n* = 31) of studies used a one-sided α level of ≤0.025; 17.7% (*n* = 33) of studies used a predefined threshold (no comparator effect size or difference between groups) to determine the sample size for efficacy. The percentage of studies with a positive authors’ conclusion but not meeting the primary endpoint, or the endpoint was equivocal, was 27.4% (*n* = 51).

**Conclusion:**

Many randomized phase II studies in oncology failed to report essential data for determining sample size calculations, many did not actually use a comparator to determine efficacy even though the studies were randomized, and many had positive conclusions even though the results were indeterminate or the primary endpoint was not met.

## Highlights

• 16.7% of randomized phase II studies used a one-sided α level of ≤0.025.
• 17.7% of studies did not use a comparator to determine efficacy or success.
• 27.4% had a positive conclusion but did not meet the primary endpoint.

## Introduction

Phase II trials are an important step in drug development because a successful trial can lead to further testing in phase III trials and/or drug approval, while an unsuccessful phase II trial may lead to a discontinuation in testing. Ideally, there would be a good balance between minimizing the premature termination of trials for potentially beneficial therapies (i.e. false negatives) and the further, costly testing of ineffective drugs (i.e. false positives).

Randomization at the phase II level has been encouraged. This trial design allows for greater assurance that the tested therapy is ‘promising’ or effective.^1^ However, even when trials are randomized, factors related to a study’s design and interpretation, besides a drug’s efficacy, influence whether a trial is viewed as successful. A prior evaluation of reporting in phase II oncology trials found that reporting is poor in many trials, which may lead to a biased interpretation.^2^

To examine the methodology and reporting of phase II oncology trials in recent years and bias in the interpretation of outcomes, we systematically reviewed current published literature.

## Materials and methods

### Search strategy

We sought to systematically assemble a list of phase II randomized oncology trials by searching PubMed with the search terms ‘oncology drug’ OR [(‘oncology’/exp OR oncology) AND (‘drug’/exp OR drug)]. We limited our search to randomized phase II clinical trials in the English language. We included all full-length articles published from 1 January 2021 to 20 June 2022 (our search date). We excluded articles that were long-term, pooled, or secondary analysis; did not include an intervention; did not test an antitumor intervention; were not randomized; were protocols only; did not include patients with cancer; were phase trials other than phase II; were reports on quality of life; were noninferiority studies; were retracted or inaccessible; were cost-effectiveness studies; or were biomarker/pharmacokinetic studies.

#### Data abstraction and variable coding

For each of the included studies, we abstracted the journal, tumor type, intervention, patients allocated to the control and intervention arm, the randomization ratio, if there was a sample size calculation, the estimated number of participants needed for the control and intervention arms based on sample size calculation, the α value and power for the sample size calculation and whether the calculation assumed a 1- or 2-sided α level, the value(s) used to determine the sample size (e.g. assumed effect size), the outcome used to determine the effect size, the primary and secondary outcomes, the results of the primary and secondary outcomes, and the authors’ conclusion of the study.

We then recoded journals to either a top journal or other, based on Google Scholar’s h5-index (≥100 versus <100) for Oncology, Hematology, and Health and Medical Science categories. (https://scholar.google.com/citations?view_op=top_venues&hl=en&vq=med_oncology) We recoded all α levels to a 1-sided value for comparability. Based on the effect size value(s) used to determine the sample size, we coded a variable to indicate a relative difference (e.g. hazard ratio), a percentage improvement based on absolute differences from prior studies, an absolute difference based off prior studies, or a predefined threshold value (e.g. a desirable threshold for continuing testing of the drug). We also compared the estimated number of participants from the sample size calculation with the number of participants allocated. If the number of participants was <10% lower in the allocated group than the estimated group, we classified the study as being underpowered; otherwise it was considered as being adequate. We classified the study conclusion as being positive, negative, or neutral, based on two blinded reviewer’s assessments. We coded each study as having met or not met the primary study endpoints, also based on two blinded reviewer’s assessments (AH and TO).

For studies that had positive conclusions but did not meet the primary study point or the endpoint result was equivocal, we coded this study as having spin. As a sensitivity analysis, we also coded spin with an expanded definition as others have done, which also considers spin when studies reported a negative overall survival secondary endpoint, but the authors’ conclusion was positive.^3^

### Determination of further testing

To differentiate phase II trials that were conducted as a step toward phase III testing or not, we used several methods: (i) we looked for discussion in the text on further/future testing in larger phase II or phase III trials, as determined by two reviewers (AH and TO); (ii) we searched PubMed to see if there were phase III trials or phase II trials with a bigger sample size carried out at a later date than the initial phase II trial, using the drug name and tumor type in the search; and (iii) we searched ClinicalTrials.gov for registered studies for the same drug and indication, using the drug name and tumor type in the search. If there was evidence of future testing for a drug in a given indication, we considered the phase II trial as being a basis for future/phase III testing. We were looking for *a priori* intent of future testing, so for negative trials, this meant that the authors concluded that there was no need for further testing. Indicating future testing based on the results of *post hoc* or subgroup analysis was not counted as having intent. Because not all studies clearly stated whether there was intent, we categorized intent as clear, vague, or none.

### Statistical analysis

To determine agreement between reviewer’s assessment, we calculated Cohen’s κ coefficients for both the study meeting its primary endpoint and the authors’ conclusion (R Statistical Software, package ‘irr’; R Foundation). Descriptive characteristics were calculated for the total sample and stratified by presence of spin. We used a Fisher’s exact test to determine an association between a study meeting its primary endpoint and the tone of the authors’ conclusion. We looked at whether there was interaction from intent to test in a phase III trial on the association between meeting the study endpoint and the tone of the authors’ conclusion, by using the Cochran–Mantel–Haenszel test. We also carried out a logistic regression analysis to see which variables were associated with questionable statistical issues, which was a dichotomous composite variable of the presence of spin, being underpowered (<90% of estimated sample size), and/or high α or low β limits. We initially included the journal impact factor, funding (industry, nonindustry, none, not indicated), year of publication, blinding status, and intent for future larger phase II or phase III studies. We removed variables if their removal resulted in a lower Akaike information criterion value. We used Microsoft Excel and R Statistical Software (version 4.2.1; R Foundation) for all analysis.

In accordance with 45 CFR §46.102(f), this study was not submitted for institutional review board approval because it involved publicly available data and did not involve individual patient data.

## Results

**Figure 1:**
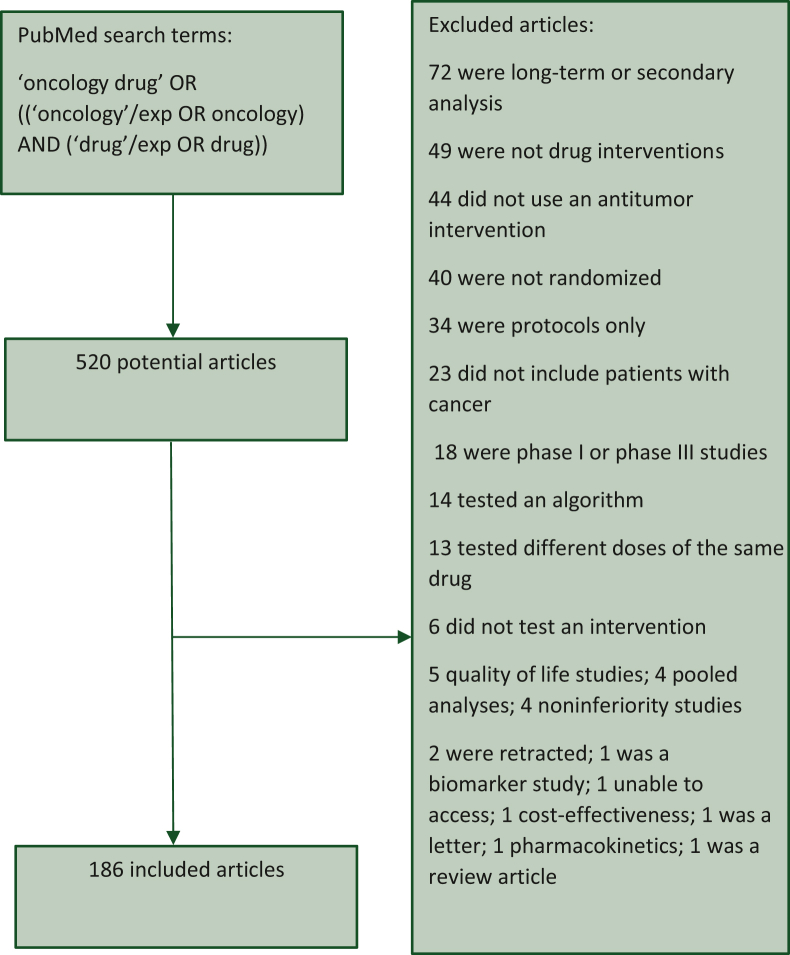
**Flowchart of search strategy for randomized phase II oncology trials**.

Our search resulted in 520 articles, of which 186 met our inclusion criteria. The flow diagram for our search strategy is in Figure 1. Of the 186 studies, the median allocated sample size was 100 (interquartile range 70-140). Most studies were open label (*n* = 153, 82.3%), and 36% (*n* = 67) were published in a top journal. Common tumor types on which studies reported were lung (*n* = 32, 17.2%), breast (*n* = 26, 14%), and gastrointestinal (*n* = 24, 12.9%). Study characteristics, by meeting the study endpoint, are presented in Table 1, and study characteristics, by intent for future testing, are in Supplementary Table S1, available at https://doi.org/10.1016/j.esmoop.2022.100779.

**Table 1:** Characteristics of randomized phase II oncology trials, overall and stratified by whether the primary endpoint was met.^a^

Characteristics	Endpoint met (*n* = 62)	Endpoint not met (*n* = 119)	Endpoint equivocal (*n* = 5)	Overall (*N* = 186)
**Total number of participants, median (IQR)**	101 (78-161)	100 (66-130)	72 (38-100)	100 (70-140)
**Phase, *n* (%)**				
I/II	2 (3.2)	1 (0.8)	0 (0)	3 (1.6)
II	58 (93.5)	115 (96.6)	5 (100)	178 (95.7)
II/III	2 (3.2)	3 (2.5)	0 (0)	5 (2.7)
**Randomization ratio, *n* (%)**				
1:1	46 (74.2)	91 (76.5)	4 (80.0)	141 (75.8)
2:1	12 (19.4)	20 (16.8)	0 (0)	32 (17.2)
Other	2 (3.2)	4 (3.4)	0 (0)	6 (3.2)
Not indicated	2 (3.2)	4 (3.4)	1 (20.0)	7 (3.8)
**Open label, *n* (%)**	50 (80.6)	98 (82.4)	5 (100)	153 (82.3)
**Top journal**^b^**, *n* (%)**	28 (45.2)	37 (31.1)	2 (40.0)	67 (36.0)
**Tumor, *n* (%)**				
Brain	1 (1.6)	5 (4.2)	1 (20.0)	7 (3.8)
Breast	6 (9.7)	19 (16.0)	1 (20.0)	26 (14.0)
Gastrointestinal	8 (12.9)	16 (13.4)	0 (0)	24 (12.9)
Hepatocellular	5 (8.1)	8 (6.7)	0 (0)	13 (7.0)
Head and neck	0 (0)	9 (7.6)	0 (0)	9 (4.8)
Leukemia	2 (3.2)	5 (4.2)	0 (0)	7 (3.8)
Lung	115 (24.2)	16 (13.4)	1 (20.0)	32 (17.2)
Myeloma	2 (3.2)	3 (2.5)	1 (20.0)	6 (3.2)
Ovarian	7 (11.3)	4 (3.4)	0 (0)	11 (5.9)
Pancreatic	3 (4.8)	8 (6.7)	0 (0)	11 (5.9)
Prostate	5 (8.1)	8 (6.7)	0 (0)	13 (7.0)
Sarcoma	1 (1.6)	5 (4.2)	0 (0)	6 (3.2)
Other	7 (11.3)	13 (10.9)	1 (20.0)	21 (11.3)
**Evidence of intent for future larger phase II or phase III trials**^c^**, *n* (%)**				
No	17 (27.4)	54 (45.4)	2 (40.0)	73 (39.2)
Yes—clear	40 (64.5)	37 (31.1)	2 (40.0)	79 (42.5)
Yes—vague	5 (8.1)	28 (23.5)	1 (20.0)	34 (18.3)

**Sample size calculations**				
**Estimated sample size, median (IQR)**	109 (82-140)	102 (80-147)	82 (56-123)	105 (80-142)
**Alpha level (1-sided), *n* (%)**				
<0.025	15 (24.2)	16 (13.4)	0 (0)	31 (16.7)
0.026-0.049	0 (0)	1 (0.8)	0 (0)	1 (0.5)
0.05	20 (32.3)	33 (27.7)	1 (20.0)	54 (29.0)
0.6-0.9	1 (1.6)	2 (1.7)	0 (0)	3 (1.6)
0.1	10 (16.1)	35 (29.4)	2 (40.0)	47 (25.3)
>0.1	8 (12.9)	8 (6.7)	0 (0)	16 (8.6)
Not indicated	8 (12.9)	24 (20.2)	2 (4.0)	34 (18.3)
**Power, *n* (%)**				
<70%	2 (3.2)	3 (2.5)	0 (0)	5 (2.7)
70%-79%	2 (3.2)	3 (2.5)	0 (0)	5 (2.7)
80%-89%	37 (59.7)	73 (61.3)	3 (60.0)	113 (60.8)
≥90%	11 (17.7)	20 (16.8)	1 (20.0)	31 (17.2)
Not indicated	10 (16.1)	20 (16.8)	1 (20.0)	34 (16.7)
**Sample size adequacy, *n* (%)**				
Adequate	48 (77.4)	76 (63.9)	2 (40.0)	126 (67.7)
Underpowered	6 (9.7)	16 (13.4)	1 (20.0)	37 (19.9)
Indeterminate	8 (12.9)	27 (22.7)	2 (40.0)	23 (12.4)
**Outcome difference for sample size, *n* (%)**				
Ratio	31 (50.0)	40 (33.6)	0 (0)	71 (38.2)
Difference	9 (14.5)	27 (22.7)	1 (20.0)	37 (19.9)
Difference+^d^	10 (16.1)	18 (15.1)	1 (20.0)	29 (15.6)
Single threshold	8 (12.9)	23 (19.3)	2 (40.0)	33 (17.7)
Not indicated	4 (6.5)	11 (9.2)	1 (20.0)	16 (8.6)

**Endpoints**				
**Primary endpoint, *n* (%)**^e^				
Disease-free survival	3 (4.8)	8 (6.7)	0 (0)	11 (5.9)
Multiple	1 (1.6)	8 (6.7)	0 (0)	9 (4.8)
Not indicated	0 (0)	0 (0)	1 (20.0)	1 (0.5)
Overall survival	5 (8.1)	16 (13.4)	0 (0)	21 (11.3)
Progression-free survival	30 (48.4)	44 (37.0)	0 (0)	74 (39.8)
Response	15 (24.2)	32 (26.9)	4 (80.0)	51 (27.4)
Safety	4 (6.5)	1 (0.8)	0 (0)	5 (2.7)
Other	4 (6.5)	10 (8.4)	0 (0)	14 (7.5)
**Endpoint reported same as powered endpoint**^f^**, *n* (%)**^e^				
Same	58 (93.5)	101 (84.9)	5 (100)	164 (88.2)
Different	1 (1.6)	2 (1.7)	0 (0)	3 (1.6)
Same but measured differently	3 (4.8)	16 (13.4)	0 (0)	19 (10.2)
**Spin, *n* (%)**				
Yes	0 (0)	46 (38.7)	5 (100)	51 (27.4)
**Authors’ conclusion, *n* (%)**				0
Positive	60 (96.8)	46 (38.7)	5 (100)	111 (59.7)
Negative	0 (0)	60 (50.4)	0 (0)	60 (32.3)
Equivocal	2 (3.2)	13 (10.9)	0 (0)	15 (8.1)

a Spin was defined as a study having a positive conclusion, but the primary endpoint was not met or the endpoint was equivocal.

b Top journal was based on Google Scholar’s h5-index (≥100) for Oncology, Hematology, and Health and Medical Science categories.

c *P* < 0.05.

d Difference+ was defined as a percentage improvement (based on absolute differences from prior studies).

e *P* < 0.001 when comparing endpoint met, not met, or equivocal.

f Underpowered was defined as a 10% lower allocation number than estimated in the sample size calculation.

The statistical power in most studies was between 80% and 89% (*n* = 113, 60.8%); 5.4% (*n* = 10) of studies used a statistical power that was inferior to 80%; and 16.7% (*n* = 34) did not indicate the level of power for the sample size calculation. 16.7% (*n* = 31) of studies used a one-sided α level of ≤0.025, 29.0% (*n* = 54) used an α of 0.05, 25.3% (*n* = 47) had a one-sided α level of 0.1, and 18.3% (*n* = 34) did not indicate the α level. Most studies (*n* = 141, 75.8%) used a 1 : 1 randomization ratio; 29% (*n* = 54) of studies did not report at least one of the elements of sample size calculations.

The median estimated sample size was 105 (interquartile range 80-142). The most common primary outcome was progression-free survival (*n* = 74; 39.8%), followed by response (*n* = 51, 27.4%) and overall survival (*n* = 21, 27.4%). Most studies (*n* = 126, 67.7%) recruited a study population size of ≥90% of the estimated sample size, but 19.9% (*n* = 37) recruited a study population size of <90% of the estimated sample size. It was indeterminate in the remaining studies.

Nearly 38% (*n* = 71) of studies used a hazard ratio from previous studies as an effect size for determining the sample size; 15.6% (*n* = 29) of studies used a predefined difference between groups, based on previous effect sizes; 19.9% (*n* = 37) of studies used prior estimates for two groups with no predefined difference between groups; and 17.7% (*n*=33) of studies used a predefined threshold (no comparator effect size or difference between groups) to determine the sample size. In 8.6% (*n* = 16) of studies, it was unclear.

The primary endpoint was met in 33.3% (*n* = 62) of studies and was negative in 64.0% (*n* = 119). The authors’ conclusion was positive in 59.7% (*n* = 111) of studies, negative in 32.3% (*n* = 60), and equivocal in 8.1% (*n* = 15) studies. The κ coefficient for the authors’ conclusion was 0.84, and the κ coefficient for a study meeting its primary endpoint was 0.93.

**Figure 2:**
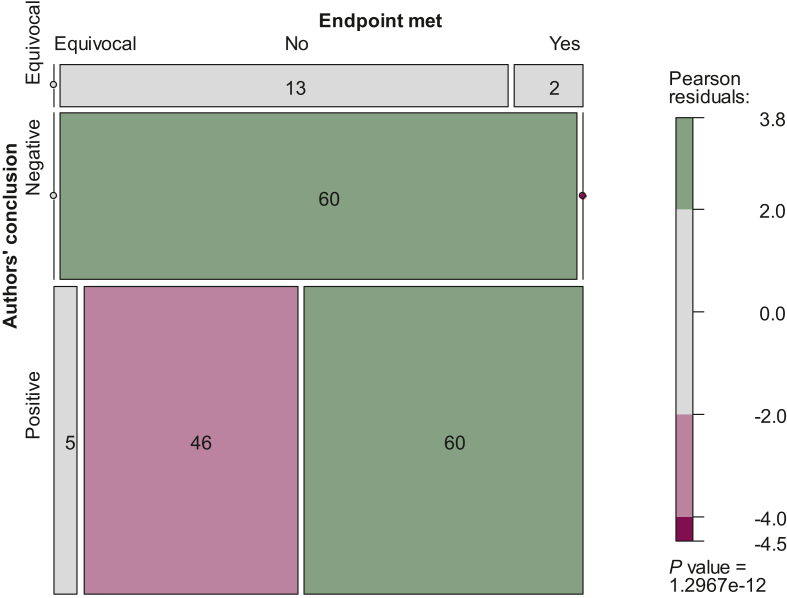
**Association between a study meeting its primary endpoint and the tone of the** authors’ **conclusion in randomized phase II oncology trials (*N* = 186).** Green indicates where the frequency is greater than expected, and red indicates where the frequency is less than expected.

The percentage of studies with spin (i.e. positive conclusion by the author but the study did not meet the primary endpoint, or the endpoint was equivocal) was 27.4% (*n* = 51). If using the expanded definition of spin, 37.1% (*n* = 69) of studies had spin. There was a strong association between a study meeting its primary endpoint and the tone of the authors’ conclusion (*P* < 0.001; Figure 2). There was significant interaction in this association by intent to publish in further, phase III trials (χ^2^ = 58.90; d.f. = 4; *P* < 0.001). In studies with clear intent for further testing, 29% of studies that did not meet the study endpoint had positive authors’ conclusions; for studies with no intent, 41% of studies not meeting the study endpoint had positive authors’ conclusions; and 73% of studies with vague intent on further testing had positive authors’ conclusions. We also noted a higher percentage of spin among studies where intent to further test was vague (50.0%), as compared with studies with clear (24.1%) or no intent (20.5%; *P* < 0.001; Supplementary Table S1, available at https://doi.org/10.1016/j.esmoop.2022.100779).

**Figure 3:**
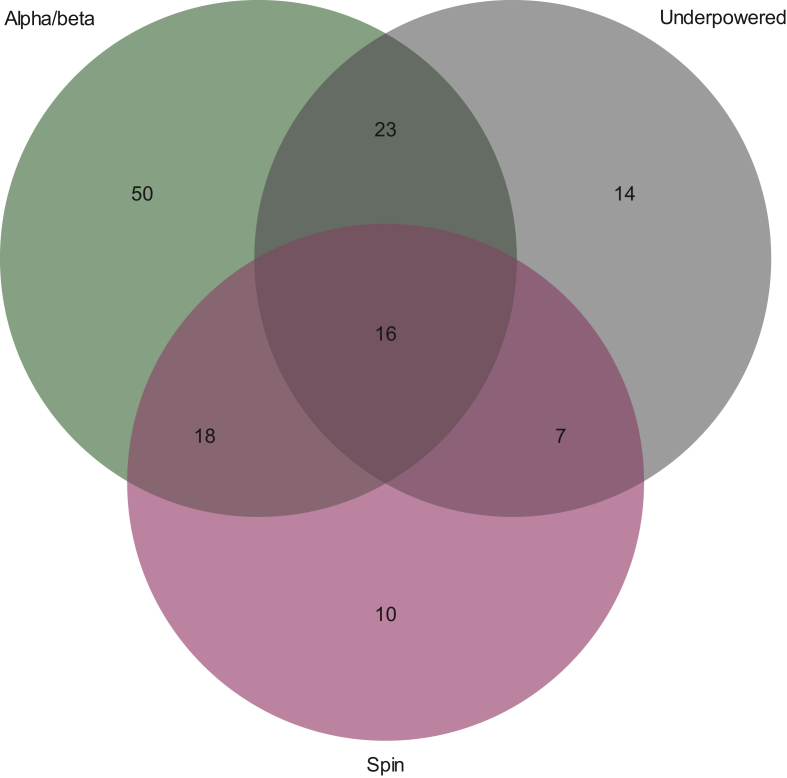
Frequency of statistical and reporting biases in randomized phase II oncology trials (*N* = 186).

We found that there were statistical or reporting issues (high α, low β, unreported α or β, underpowered, or spin) in 74.2% (*n* = 138) of studies. There were 8.6% (*n* = 16) of studies that had potential bias in all three areas (Figure 3); 21.0% (*n* = 39) with high α or low β and underpowered; 12.4% (*n* = 23) that were underpowered and had spin; and 18.3% (*n* = 34) that had high α or low β and spin.

After adjusting for journal impact factor, funding, and intent for future testing, being published in a high-impact journal (odds ratio 0.42, 95% confidence interval 0.20-0.88), compared with a non-high-impact journal, and having no funding (odds ratio 0.10, 95% confidence interval 0.01-0.66), compared with having industry funding, were both associated with lower odds of having either high α or low β limits, being underpowered, and/or having spin. We found no other variables were associated with these statistical issues.

## Discussion

We found that among phase II randomized oncology trials, about one-third (29%) failed to adequately report data on sample size calculations, and about one-fifth were underpowered. In addition, almost one-third of studies (27%) had spin, meaning they presented positive conclusions even though the results were negative or equivocal. The presence of spin was especially notable in studies where intent for further testing was vague. These findings are noteworthy given that phase III studies are often conducted based on phase II trials results. However, if the decision to pursue the drug development from phase II to phase III is based on unreliable positive findings, because of either spin in the reporting of results or spurious findings due to statistical consideration, this may result in more phase III trials being conducted unnecessarily.

When the sample size and power calculation are based on primary endpoints, this may result in inadequate sample size for nonprimary endpoints, and spurious results, including positive ones, are more likely to occur.

First, we detected spin in 27% of studies if using the conservative definition, but 37% if using a more liberal definition as others have used.^3^ This was most often because of a focus on secondary outcomes or because the determination of success was unclear (e.g. no testing between groups) and results could be interpreted subjectively. Spin in scientific publications can unfavorably lead to misinformed clinical practice guidelines or health policies, or it could lead to the implementation of health practices that are later found to be ineffective,^4^ especially because the perception of benefit among many practitioners and oncologists is influenced by the authors’ conclusion in the abstract only.^5^^,^^6^

Second, our findings raise concerns about the risk of spurious findings in phase II trials due to statistical consideration. When designing a trial, a set of statistical values is prespecified, for instance, α and power (1 – β). These prespecified values, in addition to other known or previously reported values, allow researchers to calculate the sample size. All these values are key in interpreting trial results.

The α value, or the significance level, is the probability of type I error, or false positive, meaning the risk one accepts of wrongly ‘rejecting’ the null hypothesis when it is true. *P* values and α levels are related: *P* values are interpreted based on prespecified α levels. The *P* value in a trial is the probability to obtain the observed data or more extreme data, assuming the null hypothesis were true. In other words, if a *P* value is 0.01, that means there is a 1% chance of observing the given result or more extreme results if the null hypothesis was true. The α levels are set arbitrarily, most commonly at 5% (i.e. 0.05) in biomedicine. In this hypothetical scenario, because α is set at 0.05 and the *P* value of 0.01 is lower than this threshold, you can ‘reject’ the null hypothesis and conclude a statistically significant result.

Therefore, using less stringent (higher) α levels leads to higher probability of concluding significant results while observing the same dataset. Researchers might justify a higher α level during phase II testing because the studies may be viewed as exploratory,^7^^,^^8^ but this practice may also result in spurious findings, which may allow potentially ineffective or harmful drugs to undergo further testing, with more patients being exposed to the drug. This has prompted debate about using, conversely, even lower α levels in clinical trials at-large.^9^^,^^10^

In our work, we showed that the most commonly accepted α level (of 0.025) was used in <17% of phase II trials. We found that many studies (35%) assumed a one-sided α level of >0.5, and only four studies provided justification for using an α level higher than a traditional two-sided 0.05 (all used a one-sided α level of ≥0.1). This is concerning given a high number of oncology drug being approved on phase II trial data.^11^^,^^12^

Power (or 1 – β) defines the risk of false-negative results (type 2 error) one is willing to accept when running a trial. We found that ∼5% of trials were powered at <80%. Using low power levels, by definition, reduces the probability of detecting a true effect, and may also lead to an exaggerated estimate of effect when the effect is significant.^13^Another underappreciated phenomenon induced by low power levels is the increased risk of statistically significant results not reflecting a true effect, but rather be spurious findings.^13^

Further, we found a notable number of studies lacking data for readers to assess the quality of sample size calculations or meeting study endpoint, with about one-third (34%) of studies lacking at least one basic element of the sample size calculation (α, 1 – β, one- or two-sided α, or expected outcomes for control/intervention groups). A missing α level was the most common reason for lacking sample size estimation data. Others have found even higher rates (72.1%) of data omission in phase III oncology trials when using an expanded list of criteria.^14^

We also found that a notable percentage (18%) of studies used a predefined threshold for determining effect size in the sample size calculations. This means that about one-fifth of studies did not actually use the comparator group to determine efficacy and could have been conducted without a randomized study design.

Our umbrella review of recent phase II trials found statistical concerns that may lead to spurious findings or spin in the results of 74% of them. The risk of such findings in phase II trials is the resulting justification to pursue drug development in phase III trials based on less stringent standards. An example of this is the testing of olaratumab in soft tissue sarcoma, which found a statistically significant overall survival result (a secondary endpoint) in a phase II trial with positive primary endpoint (thus based on >0.05 α level). The trial results led to olaratumab’s Food and Drug Administration approval, but the drug was later withdrawn after negative phase III results were released.

### Strengths and limitations

A strength of this study is that it is a contemporary analysis of a comprehensive list of variables related to the reporting of clinical trials. We were also able to evaluate the level of bias in the reporting of conclusions. This study did have several limitations. The categorization of spin could be somewhat subjective, but we tried to use predefined criteria, and two independent reviewers coded this variable. As there are word limits for some journals, some authors may have omitted information in the manuscript, which meant that these studies were coded as poor reporting, even though the methods could have been adequate. We also only included articles from 2021 and 2022, so our results only apply to those years. Changing methodological and reporting practices over time may mean that studies from other years could have different reporting quality. Finally, our categorization of intent for future studies may not have been correct because authors did not always report whether a future phase III trial was planned, and language describing future studies could be subjective. To allay potential bias, we used two reviewers, and if there was doubt, we gave credit to the study.

### Conclusion

We found that many randomized phase II studies in the oncology failed to report essential data for determining sample size calculations, many did not actually use a comparator to determine efficacy even though the studies were randomized, and many had positive conclusions even though the results were indeterminate or the primary endpoint was not met. Phase II trials are not usually confirmatory, and therefore may be considered somewhat exploratory, but they should still adhere to the same reporting standards and be interpreted in the context of their primary endpoint and endpoints important for the patient.
