# Supplementary material for: Design, power, and alpha levels in randomized phase II oncology trials
Source: ESMO Open. 2023 Feb 1;8(1):100779. doi: 10.1016/j.esmoop.2022.100779 (PMC10024120; doi:10.1016/j.esmoop.2022.100779)
Supplement: Supplementary — Table S1 [file mmc1.docx]

Supplemental table. Characteristics of randomized phase II oncology trials, overall and stratified by whether there was *a priori* intent for future testing

|  | **No intent**  **(n=73)** | **Clear intent**  **(n=79)** | **Vague intent**  **(n=34)** |
| --- | --- | --- | --- |
| **Total number of participants, median (IQR)** | 105 (86, 142) | 108 (80, 155) | 94 (75, 120) |
| **Phase, n (%)** |  |  |  |
| I/II | 1 (1.4) | 1 (1.3) | 1 (2.9) |
| II | 72 (98.6) | 74 (93.7) | 32 (94.1) |
| II/III | 0 | 4 (5.1) | 1 (2.9) |
| **Randomization ratio, n (%)** |  |  |  |
| 1:1 | 56 (76.7) | 58 (73.4) | 27 (79.4) |
| 2:1 | 15 (20.5) | 12 (15.2) | 5 (14.7) |
| Other | 1 (1.4) | 5 (6.3) | 0 |
| Not indicated | 1 (1.4) | 4 (5.1) | 2 (5.9) |
| **Open label, n (%)** | 58 (79.5) | 66 (83.5) | 29 (85.3) |
| **Top journal^2^, n (%)** | 24 (32.9) | 31 (39.2) | 12 (35.3) |
| **Tumor, n (%)** |  |  |  |
| Brain | 3 (4.1) | 4 (5.1) | 0 |
| Breast | 15 (20.5) | 7 (8.9) | 4 (11.8) |
| Gastrointestinal | 12 (16.4) | 10 (12.7) | 2 (5.9) |
| Hepatocellular | 5 (6.8) | 7 (8.9) | 1 (2.9) |
| Head and neck | 4 (5.5) | 3 (3.8) | 2 (5.9) |
| Leukemia | 3 (4.1) | 2 (2.5) | 2 (5.9) |
| Lung | 8 (11.0) | 16 (20.3) | 8 (23.5) |
| Myeloma | 2 (2.7) | 3 (3.8) | 1 (2.9) |
| Ovarian | 4 (5.5) | 5 (6.3) | 2 (5.9) |
| Pancreatic | 5 (6.8) | 3 (3.8) | 3 (8.8) |
| Prostate | 4 (5.5) | 8 (10.1) | 1 (2.9) |
| Sarcoma | 2 (2.7) | 3 (3.8) | 1 (2.9) |
| Other | 6 (8.2) | 8 (10.1) | 7 (20.6) |
| **Sample size calculations** |  |  |  |
| **Estimated sample size, median (IQR)** | 102 (73, 144) | 102 (69, 151) | 91 (71, 120) |
| **Alpha level (1-sided), n (%)** |  |  |  |
| <0.025 | 10 (13.7) | 13 (16.5) | 8 (23.5) |
| 0.026-0.049 | 0 | 1 (1.3) | 0 |
| 0.05 | 25 (34.2) | 20 (25.3) | 9 (26.5) |
| 0.6-0.9 | 0 | 1 (1.3) | 2 (5.9) |
| 0.1 | 19 (26.0) | 19 (24.1) | 9 (26.5) |
| >0.1 | 6 (8.2) | 9 (11.4) | 1 (2.9) |
| Not indicated | 13 (17.8) | 16 (20.3) | 5 (14.7) |
| **Power, n (%)** |  |  |  |
| <70% | 2 (2.7) | 1 (1.3) | 2 (5.9) |
| 70%-79% | 2 (2.7) | 1 (1.3) | 2 (5.9) |
| 80%-89% | 47 (64.4) | 47 (59.5) | 19 (55.9) |
| 90% or more | 12 (16.4) | 14 (17.7) | 6 (17.6) |
| Not indicated | 10 (13.7) | 16 (20.3) | 5 (14.7) |
| **Sample size adequacy, n (%)** |  |  |  |
| Adequate | 49 (67.1) | 55 (69.6) | 22 (64.7) |
| Underpowered | 18 (24.7) | 12 (15.2) | 7 (20.6) |
| Indeterminate | 6 (8.2) | 12 (15.2) | 5 (14.7) |
| **Outcome difference for sample size, n (%)** |  |  |  |
| Ratio | 32 (43.8) | 28 (35.4) | 11 (32.4) |
| Difference | 18 (24.7) | 11 (13.9) | 8 (23.5) |
| Difference+^3^ | 10 (13.7) | 16 (20.3) | 3 (8.8) |
| Single threshold | 8 (11.0) | 15 (19.0) | 10 (29.4) |
| Not indicated | 5 (6.8) | 9 (11.4) | 2 (5.9) |
| **Endpoints** |  |  |  |
| **Primary endpoint, n (%)** |  |  |  |
| Disease free survival | 5 (6.8) | 4 (5.1) | 2 (5.9) |
| Multiple | 3 (4.1) | 3 (3.8) | 3 (8.8) |
| Not indicated | 0 | 1 (1.3) | 0 |
| Overall survival | 7 (9.6) | 9 (11.4) | 5 (14.7) |
| Progression-free survival | 32 (43.8) | 32 (40.5) | 10 (29.4) |
| Response | 19 (26.0) | 21 (26.6) | 11 (32.4) |
| Safety | 0 | 5 (6.3) | 0 |
| Other | 7 (9.6) | 4 (5.1) | 3 (8.8) |
| **Endpoint reported same as powered endpoint^4^, n (%)** |  |  |  |
| Same | 69 (94.5) | 68 (86.1) | 27 (79.4) |
| Different | 1 (1.4) | 1 (1.3) | 1 (2.9) |
| Same but measured differently | 3 (4.1) | 10 (12.7) | 6 (17.6) |
| **Endpoint met, n (%)*** |  |  |  |
| Yes | 17 (23.3) | 40 (50.6) | 5 (14.7) |
| No | 54 (74.0) | 37 (46.8) | 28 (82.4) |
| Equivocal | 2 (2.7) | 2 (2.5) | 1 (2.9) |
| **Spin, n (%)*** |  |  |  |
| Yes | 15 (20.5) | 19 (24.1) | 17 (50.0) |
| **Author’s conclusion, n (%)** |  |  |  |
| Positive | 31 (42.5) | 58 (73.4) | 22 (64.7) |
| Negative | 33 (45.2) | 17 (21.5) | 10 (29.4) |
| Equivocal | 9 (12.3) | 4 (5.1) | 2 (5.9) |

*p<0.001 when comparing 3 intent for future testing groups
